# The coming era of precision medicine for intensive care

**DOI:** 10.1186/s13054-017-1910-z

**Published:** 2017-12-28

**Authors:** Jean-Louis Vincent

**Affiliations:** Department of Intensive Care, Erasme University Hospital, Université libre de Bruxelles, route de Lennik 808, 1070 Bruxelles, Belgium

## Abstract

Recent advances in technology and better understanding of mechanisms underlying disease are beginning to enable us to better characterize critically ill patients. Instead of using nonspecific syndromic groupings, such as sepsis or acute respiratory distress syndrome, we can now classify individual patients according to various specific characteristics, such as immune status. This “personalized” medicine approach will enable us to distinguish patients who have similar clinical presentations but different cellular and molecular responses that will influence their need for and responses (both negative and positive) to specific treatments. Treatments will be able to be chosen more accurately for each patient, resulting in more rapid institution of appropriate, effective therapy. We will also increasingly be able to conduct trials in groups of patients specifically selected as being most likely to respond to the intervention in question. This has already begun with, for example, some new interventions being tested only in patients with coagulopathy or immunosuppressive patterns. Ultimately, as we embrace this era of precision medicine, we may be able to offer precision therapies specifically designed to target the molecular set-up of an individual patient, as has begun to be done in cancer therapeutics.

## Background

Intensive care medicine is still a relatively young specialty but in its short lifetime has evolved rapidly with huge advances in technology and understanding of disease pathogenesis and processes. However, progress in therapeutics has been much less obvious. The fact that for decades we have enrolled heterogeneous, poorly characterized patient groups into our clinical trials goes a long way to explaining why we still have no new therapies, notably for sepsis; the sepsis response is so complex and personal that no single agent will be effective in all patients with sepsis. Now, as a result of advances in technology, greater comprehension of disease pathogenesis and pathophysiology, new understanding of biochemical and hematological data, novel genomic, proteomic, and metabolomic techniques, and improved data mining and computational modeling, we have begun to be able to characterize critically ill patients more precisely, moving beyond the global nonspecific syndromic groupings of the past (e.g., “systemic inflammatory response syndrome (SIRS)”, “sepsis”, “acute respiratory distress syndrome (ARDS)”) to more detailed classification and characterization at an individual patient level. This approach will enable us to determine on a more personal level which treatments will be best adapted to each patient, thus maximizing his/her chances of survival.

### From “poorly characterized” to “personalized” medicine

Although patients are individuals, traditionally we have tended to “label” them according to their disease or condition and often treated them accordingly, using similar interventions and therapies for all patients with the same “diagnosis”. Indeed, this has been one of the key problems with randomized controlled trials in critically ill patients—particularly those with sepsis—in which interventions have been tested in poorly characterized groups of patients believed to be similar because they meet a specific definition or have a specific diagnosis, but in fact varying markedly at an individual level with different infecting organisms, durations of disease, degrees of immune response, comorbidities, and so forth [1–3]. The results of such trials have not surprisingly been mostly negative. However, for many of these studies that showed no overall efficacy on outcome, later analyses suggested that the intervention may have been effective in specific subgroups of patients. For example, Man et al. [4] used whole genome amplification on samples from patients in the Protein C Worldwide Evaluation in Severe Sepsis (PROWESS) study [5] and identified genetic biomarkers that identified subgroups of patients with a greater response to drotrecogin alfa (activated). Similarly, Shakoory et al. [6] recently analyzed data from a randomized controlled trial of an interleukin (IL)-1 receptor antagonist that had shown no overall effect on outcome and identified a subgroup of patients with so-called macrophage activation syndrome (sepsis plus hepatobiliary dysfunction/disseminated intravascular coagulation) in which the mortality rate was significantly reduced with the intervention compared to placebo (hazard ratio for death 0.28 (95% confidence interval 0.11–0.71); *p* = 0.0071). Being able to better characterize patients will enable us to identify such subgroups, enabling interventions to be tested in more targeted populations and treatments to be personalized to a much greater extent than is currently possible.

Physicians have always characterized patients to some degree, using physical signs and physiological variables (e.g., blood pressure, heart rate, or blood glucose concentration) to diagnose and adjust aspects of management. However, these are very global measures and additional, more specific markers are needed to clearly distinguish one individual from another. Over the years, multiple biomarkers have been proposed for this purpose for various critical illness conditions, including sepsis [7], ARDS [8], and acute kidney injury [9]. However, no biomarker has been found to be adequate in terms of specificity. Indeed, individual biomarkers may be inadequate to represent these complex conditions and combinations or panels of biomarkers may be more effective. For example, Gibot et al. [10] reported that a combined score of procalcitonin (PCT), soluble triggering receptor expressed on myeloid cells (sTREM-1), and the polymorphonuclear CD64 index diagnosed sepsis better than did any of the individual biomarkers. Ware et al. [11] showed that a panel of five biomarkers for ARDS (surfactant protein-D (SP-D), receptor for advanced glycation end-products (RAGE), IL-8, club cell secretory protein (CC-16), and IL-6) could predict a diagnosis of ARDS in patients with sepsis with an AUC of 0.75. However, which biomarkers should be included in such panels remains unclear, especially as the inflammatory markers present likely vary at different time points during the disease; cost and availability are also important concerns.

Advances in genomic, proteomic, and metabolomic technology and application of these techniques to large datasets using sophisticated statistical modeling and analysis are facilitating the move toward more accurate and precise patient diagnosis and characterization. For example, Langley et al. [12] analyzed metabolomic and transcriptomic datasets from primates with *Escherichia coli* sepsis and identified a four-metabolite panel that was able to diagnose sepsis in two human cohorts with AUCs of 0.78 and 0.82, respectively. McHugh et al. [13] identified a microarray of four RNA biomarkers that predicted the presence of sepsis with an AUC of 0.88 and discriminated sepsis from infection-negative systemic inflammation better than all other tested clinical and laboratory parameters. Calfee et al. [14], using latent class data analysis, identified two subphenotypes of patients with ARDS, one of which was characterized by higher plasma concentrations of inflammatory biomarkers, greater vasopressor use, lower serum bicarbonate concentrations, and a higher prevalence of sepsis; these patients had worse outcomes and different responses to ventilator management strategies. Davenport et al. similarly identified two subphenotypes of patients with community-acquired pneumonia using a sophisticated genomic analysis. Patients with a type 1 sepsis response signature (SRS) profile had an immunosuppressed phenotype, with endotoxin tolerance, T-cell exhaustion, and HLA class II downregulation, and had higher 14-day mortality than patients with the type 2 SRS profile [15]. In children with septic shock, using whole-genome expression profiling, Wong et al. [16] identified two subphenotypes of septic shock based on a 100-gene expression signature; one of these subgroups was found to have increased mortality when prescribed corticosteroids, supporting the potential use of personalized medicine in guiding individual therapeutic decisions.

### Challenges for the coming era

We are thus moving rapidly into an era where we will be able to “personalize” treatments for individual patients [17]. But the next step, to “precision” molecular-based targeting of treatments, is much further away. Indeed, critical illness is very different to the areas in which precision medicine has made a large impact, notably oncology in which therapies are now increasingly guided by the molecular and genomic features of a tumor in a specific patient. Most oncology patients will have one tumor that can be identified and clearly characterized, enabling the most appropriate treatment to be started. Most critically ill patients have more complex, heterogeneous disease with multiple comorbidities and conditions that can impact on outcomes and response to treatment, making it difficult to identify a single target. Moreover, although tumors progress and evolve over time and treatments may need to be adapted accordingly, in general such alterations are relatively slow compared to the very rapid changes that can occur in critically ill patients. Any tests to characterize or phenotype patients therefore need to be rapidly available and repeatable. This is just one of the many challenges as we move toward personalized, and perhaps later “precision”, medicine. Here I list just a few more that I see as of key importance—there are many others.

First, there is an urgent need for international collaboration among researchers and industry to ensure standardization of measurements and reporting so that the vast amounts of data that are being generated can be compared and used together for analysis. Ideally data should be input using similar structures and systems so that they can be combined easily into single datasets and shared among all players. Increasingly, in addition to physiological and other healthcare data, “omics” data need to be routinely monitored and recorded [18]. Problems of storage for the huge databases that will be generated will need to be overcome, as will ethical issues related to patient privacy and consent.

A second challenge will be to work out how exactly the ability to characterize and subphenotype patients at a research level can be moved into the clinical arena to improve patient outcomes. Being able to better characterize patients is already being used to more carefully select for clinical trials those patients who are most likely to respond to the treatment being studied. For example, a study comparing the immunostimulating drug granulocyte–macrophage colony-stimulating factor (GM-CSF) with placebo is currently ongoing, enrolling only patients know to be immunodepressed based on their human leukocyte antigen (HLA)-DR level (ClinicalTrials.gov NCT02361528). Pharmacogenomics is widely used in some cancer therapies, but has not yet been widely studied in the ICU, partly because genomic testing is not yet available sufficiently rapidly for use in the acute critical illness situation [19]. However, genetic variations and polymorphisms have been shown to influence the response and adverse effects of several drugs relevant to the critically ill population, including morphine, dexmedetomidine, vasopressin, and catecholamines [20]. Ultimately, it is hoped that the large databases of patient information currently being collected will be used to create so-called SuperModels [21]. By inputting the present patient’s data and comparing them with the datasets already in the system, a simulated computational/mathematical model of the likely risks and therapeutic responses for that patient will be built, enabling precise preventive and/or therapeutic treatment to be given. Importantly, these complex models will need to be able to capture and predict the temporal and dynamic variability of critical illness [22]. Continuing data input into intelligent models will enable increasingly precise models to be developed, facilitating the translation to clinical reality.

The economic challenge of personalized medicine is unknown and impossible to predict. Although the costs of genomic analyses are currently high, prices will fall as these tests are more widely used and available. New drug development is expensive, but the improved knowledge of the underlying molecular mechanisms of disease provided by the advances discussed and the ability to more accurately target those patients most likely to respond to a new therapy may make drug development more efficient, thus potentially reducing costs. Precise knowledge of the most appropriate therapy for each patient and better preventive therapy will reduce unnecessary therapies and costly adverse drug reactions. Although costs are thus likely to be increased in the initial years, this is expected to be balanced by more accurate and efficient patient management.

## Conclusion

The personalized medicine approach encourages us to develop a more singular approach to patients, treating each individual according to their specific history, characteristics, and ongoing needs. Treatment prescriptions will be (are already being) more accurately targeted at each individual’s specific phenotype, resulting in more effective therapy and improved outcomes. Treating individuals rather than diseases will necessitate a paradigm change in our approach to diagnosis *and* management. Clinicians, researchers, and industry must all work ogether to embrace the promises and potential of this exciting new era.
